# Investigating Cortical Buffering Effects of Acute Moderate Intensity Exercise: A cTBS Study Targeting the Left Dorsolateral Prefrontal Cortex

**DOI:** 10.3389/fnhum.2021.645326

**Published:** 2021-09-30

**Authors:** Felicia Manocchio, Cassandra J. Lowe

**Affiliations:** ^1^School of Public Health Sciences, University of Waterloo, Waterloo, ON, Canada; ^2^Brain and Mind Institute, Western University, London, ON, Canada; ^3^Department of Psychology, Western University, London, ON, Canada

**Keywords:** TBS, rTMS (repetitive transcranial magnetic stimulation), exercise, DLPFC (dorsolateral prefrontal cortex), cortex, health

## Abstract

**Background:** The beneficial effects of both single-session bouts of aerobic exercise and therapeutic exercise interventions on the cortical regions associated with top-down attentional control [i.e., prefrontal cortex (PFC)] have been well documented. However, it remains unclear whether aerobic exercise can be used to buffer against suppressive influences on the dorsolateral PFC (dlPFC).

**Objective:** The current study sought to determine whether a single session of moderate intensity aerobic exercise can offset the expected suppressive effects of continuous theta burst stimulation (cTBS) targeting the dorsolateral prefrontal cortex (dlPFC).

**Methods:** Twenty-two right-handed participants (aged 19–30) completed a 20-minute movement-only control session [10% heart rate reserve (HRR)] and moderate intensity (50% HRR) exercise in a counterbalanced order. Following each exercise session, participants received active cTBS to the left dlPFC. Changes in executive functions were quantified using a Flanker paradigm employed at baseline, post-exercise and post-cTBS time points. Additionally, EEG was used to measure changes in event-related potential components related to inhibitory control (i.e., N2) and attentional control (i.e., P3) during the flanker task.

**Results:** Behavioral results from the flanker task revealed a significant improvement in task performance following an acute bout of moderate intensity exercise. Furthermore, the effect of cTBS in both the movement-only control and moderate intensity conditions were non-significant. Similarly, EEG data from P3b and N2 ERP components revealed no changes to amplitude across time and condition. P3b latency data revealed a significant effect of time in both the moderate intensity and movement-only conditions, such that P3b latencies were significantly shorter across time points. Latency data within the N2 ERP component revealed no significant interactions or main effects.

**Conclusion:** The findings of the current study provide tentative support for the hypothesis that both moderate and light intensity exercise promote cortical buffering against the suppressive effects of cTBS targeting the dlPFC. However, in the absence of a no-movement control, a lack of expected suppressive effects of cTBS cannot be ruled out.

## Introduction

Across the lifespan, executive functions are a robust predictor of several psychosocial, intellectual, and health-related outcomes, including academic performance, adherence to health protective behaviors, and substance use and abuse (Romer et al., 2009; Moffitt et al., 2011; Kim and Lee, 2011; Papsideris et al., 2020). Indeed, the consistent implementation of everyday behaviors, such as dietary self-regulation, medication adherence, and academic performance (Insel et al., 2006; Diamond, 2013; Lowe et al., 2018a), are typically thought to be dependent on executive functions, and by extension the underlying cortical substrates (i.e., the prefrontal cortex). Although executive functions are relatively stable within individuals, several naturally occurring variables can induce state-like fluctuations in prefrontal cortical activity, leading to discernable reductions in cognitive control. These modulators include, but are not limited to; sleep restriction, acute stress, and alcohol intoxication (Arnsten, 2009; Brondel et al., 2010; Marinkovic et al., 2012; Schulte et al., 2012; Lowe et al., 2017). Overtime, these fluctuations can have a profound impact on individual and societal health and well-being, highlighting the need for targeted interventions aimed at offsetting state-like fluctuations in executive control.

Broadly conceived, the term “executive function” is used to denote a set of higher order cognitive processes that play a critical role in top-down control and goal-directed behaviors (Baddeley, 1996; Miyake et al., 2000); these processes are distinguishable from other crystalized forms of mental activity. Constituent regions within the executive control network, including the prefrontal cortex (PFC), posterior parietal cortex, inferior frontal junction, and anterior insula comprise the neuroanatomical regions typically associated with executive functioning (Miller, 2000; Miller and Cohen, 2001; Yuan and Raz, 2004; Derfuss et al., 2005; Alvarez and Emory, 2006; Niendam et al., 2012; Hutchison and Morton, 2016). Within this network, the PFC, particularly the dorsolateral prefrontal cortex (dlPFC), is thought to be a neuroanatomical region that is strongly implicated in executive function. Hemispheric lateralization within the PFC is thought to contribute to distinct facets of response inhibition and working memory (Barbey et al., 2013). Specifically, left hemisphere activation is often associated with tasks involving the top-down control of attentional tasks (Vanderhasselt et al., 2009), and verbal working memory demands (Wager and Smith, 2003), whereas motor response inhibition (Aron et al., 2014), visual-spatial working memory (Smith et al., 1996; Reuter-Lorenz et al., 2000) and macro-adjustments of cognitive control (Vanderhasselt et al., 2009) appear to be more dependent on right hemisphere activation.

Given the association between dlPFC functionality and executive functions, targeted interventions aimed at enhancing dlPFC functionality can potentially support and maintain optimal performance. Such interventions may be especially pertinent in reducing the impact of acute modulators on executive control. Along these lines, a growing body of evidence has demonstrated that both long-term randomized interventions and acute bouts of aerobic exercise can improve executive function task performance (Smith et al., 2010; Chang et al., 2012b; Chaddock-Heyman et al., 2013; Ludyga et al., 2016). These effects are observed following as little as 20 min of moderate intensity aerobic exercise, with the largest effects being observed 11-20 min post-exercise (Chang et al., 2012a); effects remain apparent for up to 50 min post-exercise (Joyce et al., 2009).

Although, a general improvement in cognitive functioning is observed following acute bouts of aerobic activity, the largest effects are observed on measures of executive functions (McMorris and Hale, 2012), indicating that executive functions may be the most sensitive to exercise-induced improvements in neurocognitive functioning. Within the acute exercise and executive function literature, the majority of studies have focused on the behavioral inhibition subcomponent. Collectively, these studies have demonstrated that a bout of acute aerobic exercise can improve task performance on measures of inhibitory control in children, young adults and older adults (Kamijo et al., 2009; Barella et al., 2010; Yanagisawa et al., 2010; Chang et al., 2011, 2012b, 2015; Hyodo et al., 2012, 2016; Lowe et al., 2014, 2016, 2017; Samani and Heath, 2018). Taken together, the available evidence suggests that acute bouts of aerobic exercise can facilitate improvements in inhibitory control.

The incorporation of neuroimaging and neurophysiological methods into exercise studies have provided important insight into the neural processes underlying the observed exercise-induced enhancements in executive functions. For instance, fMRI studies have demonstrated that higher levels of fitness are associated with larger hippocampal volumes, and as a result superior relational memory (Chaddock-Heyman et al., 2010). Additionally, increased resting state functional connectivity between the dlPFC and superior parietal gyrus is observed following aerobic exercise (Prehn et al., 2017). Further, Electrophysiological studies, have demonstrated that the latency and amplitude of the P3b and N2 event related potential (ERP) components are especially sensitive to aerobic exercise protocols. While the P3b is typically regarded as reflecting the allocation of attention resources, the N2 is more often regarded as a measure of conflict monitoring and response inhibition (Polich, 2007; Folstein and Van Petten, 2008). More specifically, the P3b is an endogenous component generated by an anatomically diffuse network of cortical and subcortical structures. This component is thought to reflect the neural processes underlying several higher cognitive functions typically engaged during executive control tasks (e.g., inhibition, working memory, attention, and stimulus processing; Polich, 2007). The amplitude of the P3b is thought to reflect the neural or attentional resources afforded to a given task or stimulus, whereas, the latency is thought to reflect processing speed (Polich, 2007).

The functionally distinct N2 component is commonly observed in conjunction with the P3b (Folstein and Van Petten, 2008). The N2 is a frontocentral component thought to be generated by medial-frontal and latero-frontal neuroanatomical regions (Karch et al., 2010). The N2 amplitudes are thought to represent the capacity to respond to errors and otherwise exert cognitive control during the early stages of response inhibition (Veen and Carter, 2002; Folstein and Van Petten, 2008; Tillman and Wiens, 2011). Thus, the N2 amplitude is larger (i.e., more negative) when an individual exerts inhibitory control (e.g., for incongruent compared to congruent trials; Kopp et al., 1996; Heil et al., 2000; Yeung et al., 2004; Bartholow et al., 2005; Tillman and Wiens, 2011).

Across a variety of cognitive tasks, greater amounts of physical activity are associated with larger P3b amplitudes and shorter latencies, indicating that the habitual engagement in physical activity has beneficial effects on neurocognitive functioning that manifests as increased attentional control and faster processing speeds (Polich and Lardon, 1997; Hillman et al., 2004, 2005; Pontifex et al., 2009). Such physical activity-related modulation of cortical functionality is substantially larger for cognitive measures requiring greater amounts of executive control (Hillman et al., 2006). Similarly, exercise-induced enhancements in the P3b amplitude (Hillman et al., 2003, 2009; Kamijo et al., 2009; Drollette et al., 2014; Chang et al., 2017) and reductions in P3b latencies (Hillman et al., 2003; Kamijo et al., 2009; Drollette et al., 2014; Chang et al., 2017) are observed following acute bouts of aerobic exercise, and these effects are specific to task components modulating inhibitory control demands. Together, these data suggest that aerobic exercise generally improves neurocognitive functioning by increasing inhibitory and attentional control abilities, and cognitive processing speed during stimulus encoding.

Exercise effects on the N2 ERP are however somewhat less well understood. While there is evidence to suggest that acute aerobic exercise is associated with smaller N2 amplitudes and shorter latencies, indicating greater inhibitory control during cognitive tasks (Pontifex and Hillman, 2007; Drollette et al., 2014), other studies have demonstrated no such effects of exercise on the N2 ERP component (Themanson and Hillman, 2006; Winneke et al., 2019). These inconsistencies across published studies warrant further investigation into the effects of exercise on the N2 ERP.

In a novel line of inquiry, Lowe et al. (2017) demonstrated that aerobic exercise has the capacity to promote cortical resilience (i.e., the ability of cortical brain regions to recover efficiently following a transient suppressive influence (Hall et al., 2020) in healthy young adults. In this study, inhibitory repetitive transcranial magnetic stimulation [rTMS; specifically, continuous theta burst stimulation (cTBS)] was used to temporarily attenuate cortical activity in neuronal populations underlying the left dlPFC prior to an acute bout of exercise. The application of cTBS induces temporary suppression of cortical excitability for up to 50 min following stimulation (Huang et al., 2005; Wischnewski and Schutter, 2015), and reliably disrupts task performance on attentional-control tasks such as the Flanker (Lowe et al., 2018b). In the present protocol, cTBS was followed with either an acute bout of moderate intensity aerobic exercise (50% heart rate reserve) or a movement-only control session (walking at 10% heart rate reserve). Results indicated that dlPFC function recovered at a significantly faster rate in the moderate intensity relative to the movement only condition. Specifically, at the 40-min post-stimulation point, 101.3% of the attenuation in Stroop performance was recovered in the moderate exercise group, but only 18.3% in the light intensity exercise control group, demonstrating that aerobic exercise can promote cortical resilience. While these findings have important theoretical and empirical implications for how we conceptualize the neuroprotective effects of exercise and supports the use of therapeutic approaches to improving cognitive function.

Currently, it remains unclear whether acute exercise can also blunt a suppressive influence in an *a priori* manner (i.e., act as a buffer against suppressive influence). The purpose of the current study was to test this latter possibility. Specifically, it was hypothesized that moderate intensity exercise would reduce cTBS-induced suppression of dlPFC function, compared to movement only control condition. In order to quantify these effects, we examined both behavioral indicators of top-down attentional control, such as Flanker performance and event related potential components indicative of the same (e.g., P3b, and N2). We hypothesized that Flanker task accuracy and reaction time would improve following moderate intensity exercise, and demonstrate buffering effects against cTBS induced attenuation. Additionally, we anticipated the P3b ERP component to improve; such that P3b amplitude would be larger, and P3b latencies would be shorter following moderate exercise, and would not be significantly altered following cTBS. Further, due to the uncertainty around exercise effects of N2 ERP, we examined it in an exploratory manner.

## Methods

A total of 23 healthy young adults participated in this study. However, 1 participant was unable to complete the second session, as they did not bring appropriate shoes; they were wearing high-heel shoes and did not have running shoes. As such, their data was excluded from all analyses, resulting in a final sample of 22 participants, aged 19 to 30 years old (see Table 1). We determined the sample size using G^∗^Power (version 3.1.9; Faul et al., 2009). The results suggest that 20 participants are required to achieve cTBS suppression effect similar to the one found in previous work (*d* = 0.76; Lowe et al., 2017), with 95% power. Participants were recruited from undergraduate courses using an online recruitment system, and from recruitment posters located around the university campus. All participants indicated that they were naïve to TMS, right-handed, and had normal or corrected-to-normal vision. Participants were financially compensated for their participation. Study procedures were approved by the University of Waterloo Research Ethics Committee and conformed to the ethical standards outlined the Declaration of Helsinki. Informed consent was obtained from all participants prior to the start of the study protocol.

**TABLE 1:** Participant characteristics.

	Mean (SD)	Range	*n* (%)
Age	21.68 (2.89)	19-30	
Sex			
Female			13 (62%)
Male			8 (38%)
Not Reported			1 (4.5%)
Race/Ethnicity			
Caucasian			11 (55%)
Asian			7 (35%)
Multiracial/other			1 (4.5%)
Declined to respond			3 (15%)
Height (m)	1.65 (0.09)	1.57 – 1.83	
Weight (kg)	61.4 (15.8)	41.7 – 86.2	
Predicted hours of aerobic exercise in next week	4.5 (2.9)		
Declined to respond	4 (18%)		
Confidence in ability to exercise daily	67% (25.9)		
Declined to respond	4 (18%)		

Prior to participation, participants were screened to be free of any neurological or psychiatric, and physical health conditions that may increase the risk of an adverse reaction to either the TMS or aerobic exercise protocol. Participants were excluded from the study if they had (a) been diagnosed with a neurological or psychiatric condition (i.e., epilepsy, depression, anxiety), (b) being treated with any psychiatric medications; (c) had a family history of epilepsy or hearing loss; (d) history of head trauma (i.e., concussion); (e) experienced chronic headaches or migraines; (f) has metal in the cranium and/or any implanted electronic or medical devices (i.e., electronic pacemaker, implanted medication pump); (g) were pregnant; (h) answered “yes” to any of the questions of the Physical Activity Readiness Questionnaire (PAR-Q).

### Procedure

A within-subject study design was utilized, such that participants completed both the moderate intensity exercise and the movement-only control conditions in a counterbalanced order; exercise sessions were separated by a one-week intersession interval. Study sessions were identical, with only the exercise intensity varying.

**Appendix Figure 1:**
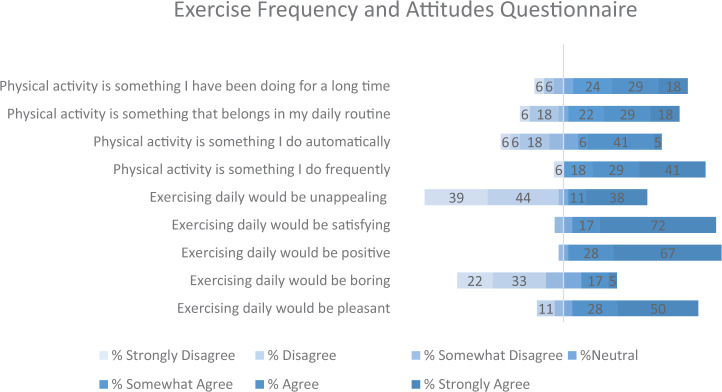
Exercise frequency and attitudes across participants. Values reflect percentage of participants that responded to each option.

**Appendix Figure 2:**
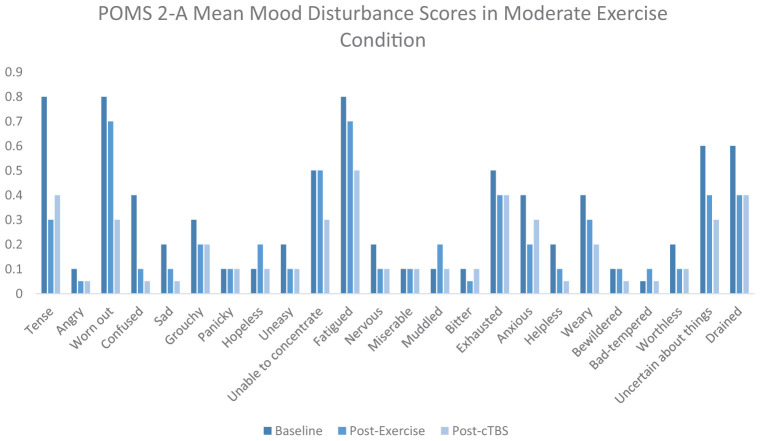
POMS 2-A mean mood disturbance scores in the moderate exercise condition.

**Appendix Figure 3:**
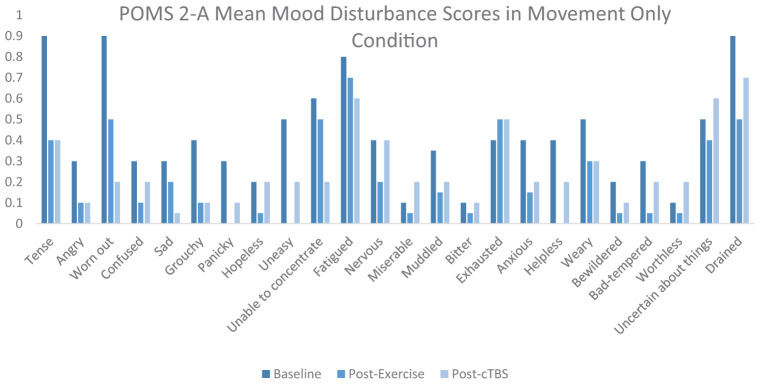
POMS 2-A mean mood disturbance scores in movement only condition.

Upon arrival, participants were fitted with the EEG cap. Next, they were asked to complete the Profile of Mood States-2 Adult Short (POMS-2A; Heuchert and McNair, 2014), which is a widely validated self-reported assessment of transient mood states. The POMS 2-A was employed as an exploratory measure of mood disturbance fluctuations across time points and conditions. Following the POMS 2-A, participants were asked to complete flanker task. In both conditions, participants walked on the treadmill for a duration of 20 min (see Appendix Figures 2, 3). As Chang et al. (2012a) found that the greatest positive exercise effects manifest after a brief rest period, participants completed the flanker task following a 10 min period of seated rest. Next, cTBS was applied over the left dlPFC, and then participants completed the flanker task (post-cTBS) again. At the end of the second study session participants were asked to complete a series of questionnaires pertaining to demographics, physical activity patterns, and dietary habits. Physical activity frequency patterns were assessed using a rating scale, responding to statements of exercise frequency. These data are presented in Appendix Figure 1. Physical activity attitudes were assessed using a rating scale pertaining to enjoyment levels of daily exercise. Dietary habits were assessed using a food frequency questionnaire, aimed at measuring participants’ regular food choices, dietary self-control and attitudes toward diet.

### Flanker Paradigm

A modified version of the Eriksen flanker task (Eriksen and Eriksen, 1974) was used to quantify exercise and cTBS effects on top-down attentional control, and to elicit ERPs. The Flanker task is a widely validated measure of executive function, and has been employed in many exercise related studies (Hillman et al., 2006; Kamijo et al., 2007; Lowe C. et al., 2018a). The version of the Flanker paradigm used in the current study was programmed in E Prime (Psychology Software Tools, Inc.), and has been previously used in the Lowe et al. (2018a) study. In the context of the Flanker paradigm, top-down attentional control is indicated by the ability to recognize and select the correct stimulus while disregarding distracting or incorrect stimuli presented at the same time. In this version, a fixation cross followed by a five-letter string, composed of Hs and/or Ss, was presented centrally on a computer screen. Participants were instructed to indicate whether the center target stimulus among four identical congruent (i.e., HHHHH or SSSSS) or incongruent (i.e., HHSHH or SSHSS) distractors was an “S” or an “H” as quickly and accurately as possible. Responses were made using the associated keys on a keyboard using their dominant hand. For each trial, the flanker stimulus was presented for 500ms, followed by a random intertrial interval (ITI) jittered between 600 and 1000 ms, with a total response window of 1000ms. Participants completed a total of 240 trials with equiprobable congruency, such that half the trials consisted of congruent stimuli (120 trials) and the other half incongruent stimuli (120 trials). The primary dependent measures were task accuracy, and the flanker interference score (reaction time on correct incongruent trials minus correct congruent trials); higher interference scores are indicative of poorer top-down attentional control.

Prior to participation in the study, participants were asked to wear appropriate exercise apparel (i.e., running shoes) and were given a bottle of water during each exercise session. Participants were fitted with a Polar FT1 heart rate monitor, and resting heart rate (RHR) was measured prior to exercise. For both the moderate exercise and movement-only control condition, participants were asked to walk on a treadmill for 20 minutes including warm up time. Speed and incline were gradually adjusted for the first two minutes until participants reached their target heart rate (THR), determined using the Karvonen formula and heart rate reserve (HRR; the difference between the age-predicted heart rate max [220-age] and resting heart rate). To ensure that all participants could complete the 20 min exercise session at a brisk walking pace, 50% HRR was selected for the moderate intensity condition. The movement-only control condition was set to 10 percent HRR, representing minimal aerobic demands during otherwise identical physical movement in relation to the moderate exercise condition. Following exercise, participants were given a 10-minute seated rest period, during which they completed the POMS 2-A mood questionnaire.

### Theta Burst Stimulation Procedure

Continuous TBS was administered using a 75 mm outer diameter figure-8 coil (MCF-B65) connected to a MagPro (model X100) stimulation unit (Medtronic, Minneapolis, MN, United States). Coil positioning was guided using a computerized frameless stereotaxic system and neuronavigation software (*Brainsight TMS*, Rogue Research, Montreal, Canada) in conjunction with an T1 weighted structural MRI scan, normalized to MNI space, from a previous data set (the same scan was used for all participants). The left dlPFC was located using the International 10-20 system (Herwig et al., 2003). Consistent with prior work (Bolton and Staines, 2011; Lowe et al., 2014, 2017, 2018a), cTBS – a 40 s continuous train consisting of 600 pulses applied in the theta burst pattern (bursts of three stimuli at 50 HZ repeated at 5 Hz frequency (Huang et al., 2005) – was administered over the left dlPFC by positioning the coil at a 90° angle from the mid-sagittal line with its center positioned over F3. Stimulation intensity was set at 80% resting motor threshold (RMT). RMT was defined at the lowest stimulation intensity required to produce a motor-evoked potential (MEP) with a peak-to-peak amplitude exceeding 50 μV in at least 5 out 10 consecutive trials. Stimulation was applied over the contralateral motor cortex, at a 45° angle tangentially to the scalp, with the handle pointing posteriorly. For each study session, individual RMTs were determined using electromyography measured from the right *abductor pollicus brevis* (APB) muscle.

Average cTBS stimulation intensity (% stimulator output) for the moderate intensity exercise and movement-only control conditions were 53.41(*SD* = 6.57) and 53.40 (5.63) respectively. No significant differences in cTBS stimulation intensity between exercise conditions was observed (*t*(21) = 0.012, *p* = 0.990,95% CI [−1.58, 1.60]).

### EEG Recording and Analysis

Continuous EEG data were recorded from 10 midline and frontal electrode sites [FP1, FP2, FPz, Fz, F3, F4, FCz, Cz, CPz, Pz] using a 64 Ag/AgCI electrode Neuroscan Quick-Cap (Compumedics, Charlotte, NC), referenced online to a mid-line electrode located between Cz and CPz and grounded to AFz. Amplitudes and latencies for the N2 component were measured from electrode sites Fz, FCz, and Cz, and were averaged together to create a frontocentral N2 cluster. The P3b component were measured from central parietal sites Cz, CPz, and Pz, and were averaged together to create a central parietal P3b cluster. Data were sampled and digitized at a rate of 1000 Hz with a bandpass filter of.1 to 70 Hz. All channel recordings had impedance values below 5 kΩ, and impedance was monitored before and after cTBS and exercise.

Offline, data were pre-processed using MATLAB 2018a and the EEGLAB toolbox (version 14; Delorme and Makeig, 2004). EEG data were filtered using a 50 HZ low-pass filter to remove signal drifts and line noise, and re-referenced to the averaged linked mastoids (M1, M2). Stimulus-locked ERP segments spanning −100 to 800 *ms* post-stimulus onset for correct congruent and incongruent trials were computed individually for each participant. The resulting data were individually decomposed using temporal independent component analysis (ICA) with extended infomax algorithm (Bell and Sejnowski, 1995; Delorme et al., 2007). Independent components that were not located within the cortex, as well as those components elicited by spurious movement and ocular artifacts were removed. Afterward, data were visually inspected, and residual non-stereotyped artifacts were removed (Mennes et al., 2010; Jiang et al., 2019).

For all dependent measures [incongruent, congruent], data were averaged relative to a 100 *ms* pre-stimulus baseline. As recommended by Luck and Gaspelin (Luck and Gaspelin, 2016), measurement time windows and electrode regions of interest for each component were determined *a priori* using typical electrode sites and time windows from prior studies. This reduces type 1 error and the bias toward significance. Electrode sites and measurement time windows were confirmed via visual inspection of the grand average waveforms. Data for all components was extracted from midline electrode sites Fz, FCz, CZ, and Pz.

Stimulus-locked amplitude and latency measures for each ERP component was calculated by determining the peak amplitude (μV) for correct congruent and incongruent flanker trials within two-time windows. The P3b was defined as the peak amplitude between 300-600ms post-stimulus presentation. Likewise, the flanker N2 was defined as a negative deflection peaking between 200 and 300 ms. As recent evidence has suggested that averaging ERP amplitudes across several electrodes to increases signal reliability (Huffmeijer et al., 2014). As such, amplitude and latency data were averaged across electrode regions of interest for each component. Specifically, a P3b cluster was created by averaging the peak amplitude (μV) and latency across central and parietal electrodes (Cz, CPz, Pz). The P3b is typically maximal at central parietal sites (Polich, 2007). The amplitudes and latencies from frontocentral electrode sites (Fz, FCz Cz) were averaged together to create a frontocentral N2 cluster.

### Data Analytic Approach

Accuracy was calculated as the proportion of correct responses to congruent and incongruent trials. Prior to analyses, reaction times less than 100 ms and greater than 3 standard deviations from the individual mean reaction time were excluded. Frequentist analyses were conducted using SPSS (version 25; IBM Corp, Armonk, NY, United States). First, paired sample *t*-tests were conducted to ensure baseline comparability between conditions in Flanker task performance and cTBS stimulation intensity. Behavioral data were analyzed using a 2 × 3 mixed ANOVA with exercise condition [moderate, active-control] and time [baseline, post-exercise, post-cTBS] as the within subject factor, and order of the exercise condition as the between subject factor. Significant interactions were followed up with simple effects one-way ANOVAS at each level of exercise condition. For significant effects, Fisher’s least square differences (LSD) *post hoc* tests were performed. Electrophysiological data were analyzed using separate 2[moderate, very-light] × 3 [baseline, post-exercise, post-cTBS] × 2 [incongruent, congruent] repeated measures ANOVAs for N2 amplitude, N2 latency, P3b amplitude, P3b latency. Significant three-way interactions were followed up with a 3 [baseline, post-exercise, post-cTBS] × 2 [congruent, incongruent] repeated measures ANOVAs at each level of exercise [moderate, very-light]. Significant interactions were followed up with simple effect ANOVAs. For significant effects, Fisher’s LSD *post hoc* tests were performed.

To account for the small sample size, we also employed a Bayesian approach. Bayesian inference is a model comparison approach that is better suited to accommodating small sample sizes than standard frequentist approaches. Bayesian statistics provide information on likelihood and strength of an effect by evaluating how well the data support the alternative over the null hypothesis. As such, this approach is capable of distinguishing between lack of power and/or precision, and the lack of an effect. Specifically, the Bayes factor quantifies how likely the alternative is relative to the null model. For instance, a Bayes factor of 10 (BF_10_) would suggests strong evidence indicating that the alternative model is 10 times more likely to occur than the null. Bayesian analyses were conducted using the default priors for *t*-test and ANOVA analyses (Rouder et al., 2012) and JASP software using the same procedures described above.

### Results

#### Baseline Comparability of Condition

No significant differences in baseline interference scores were observed (*t*(21) = 0.904, *p* = 0.376, 95% CI [−6.99, 17.74]; BF_10_ = 0.321), indicating comparable baseline performance between movement-only control and moderate exercise conditions. Likewise, no significant differences in cTBS stimulation intensities between exercise conditions were observed (*t*(20) = 0.01, *p* = 0.990, 95% CI [−1.58, 1.60]; BF_10_ = 0.228

#### Behavioral Results

Performance accuracy, reaction times on incongruent and congruent trials, and flanker interference scores as a function of exercise condition (active-control or moderate intensity exercise) and time (baseline, post-exercise, post-cTBS) are presented in Table 2. Effect size was quantified by Cohen’s (*d*).

**TABLE 2:** Accuracy, reaction time (RT) (ms), and interference scores (ms) across time and conditions.

	Moderate Exercise Mean (SD)			Movement-only Control Mean (SD)		
	Baseline	Post-Exercise	Post-cTBS	Baseline	Post-Exercise	Post-cTBS
Accuracy Congruent	0.91 (0.08)	0.90 (0.12)	0.89 (0.12)	0.89 (0.12)	0.90 (0.13)	0.90 (0.12)
Accuracy Incongruent	0.84 (0.11)	0.83 (0.13)	0.83 (0.13)	0.80 (0.15)	0.84 (0.15)	0.86 (0.14)
Congruent RT	193.16 (59.05)	197.16 (61.29)	188.12 (52.48)	183.47 (73.68)	179.10 (62.10)	174.72 (57.62)
Incongruent RT	221.11 (55.25)	207.66 (59.45)	202.64 (52.28)	206.05 (68.51)	202.89 (64.63)	204.07 (59.58)
Flanker Interference	27.95 (13.21)	10.51 (13.79)	14.51 (17.63)	22.58 (24.27)	23.78 (27.03)	29.35 (23.01)
Exercise Effect	−16.67 (15.71)			1.20 (17.59)		
cTBS Effect		4.01 (16.74)			5.57 (21.88)	

Interference score analyses indicated that the order by exercise condition (*F*(1, 20) = 2.15 *p* = 0.158, *d* = 0.655), order x time (*F*(2,40) = 0.73, *p* = 0.489, *d* = 0.369), and the three-way [order × time × exercise condition] interaction (*F*(1, 20) = 0.69, *p* = 0.507, *d* = 0.381) were not significant. Although the main effect of exercise condition was not significant (*F*(1, 20) = 2.73, *p* = 0.114, *d* = 0.739), a significant main effect of time (*F*(2, 40) = 5.43, *p* = 0.008, *d* = 1.04) was observed. This was qualified by a significant time [baseline, post-exercise, post-cTBS] by exercise condition [moderate, movement-only control] interaction (*F*(2, 40) = 5.86, *p* = 0.006, *d* = 1.08).

**FIGURE 1:**
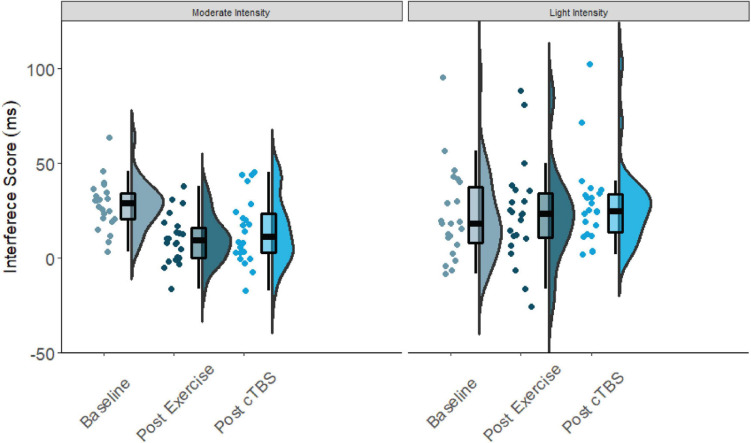
Flanker interference scores at baseline, post-exercise, post-cTBS in moderate intensity exercise and movement only conditions.

**FIGURE 2:**
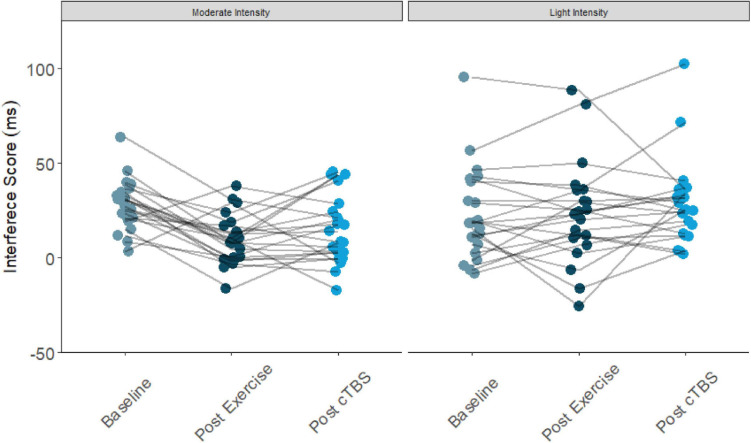
Flanker interference scores at baseline, post-exercise, post-cTBS in moderate intensity exercise and movement only conditions.

Results from the one-way simple main effects ANOVA indicated a significant main effect of time for the moderate intensity exercise condition (*F*(2, 42) = 12.23, *p* < 0.001, *d* = 1.526). Planned LSD comparisons revealed that flanker interference scores were significantly lower (*p* < 0.001, 95% CI [10.00, 24.88) following the acute bout of moderate aerobic exercise relative to baseline. No significant differences in post-exercise and post-cTBS interference scores were observed (*p* = 0.274, 95% CI[−11.43, 3.41). Most notably, post-cTBS interference scores were significantly lower than baseline scores (*p* = 0.003, 95%CI [5.27, 21.60]), indicating a potential buffering effect of aerobic exercise; see Figures 1, 2. The main effect of time was not significant for the movement-only control condition (*F*(2,42) = 1.25, *p* = 0.298, *d* = 0.487).

Further, accuracy analyses indicated a significant main effect of congruency (*F*(1, 20) = 35.31, *p* < *0.001*, *d* = 2.62), such that flanker trials were significantly more accurate on congruent trials compared to incongruent trials. No significant main effects of time (*F*(1, 20) = 0.65, *p* = 0.53, *d* = 0.36), or exercise condition (*F*(1,20) = 0.043, *p* = 0.837, *d* = 0.09) were observed. Additionally, no significant interactions (*p* > 0.12) were observed.

### Bayesian Statistics

Results from the 2 × 3 Bayesian repeated measures ANOVA analyses revealed anecdotal evidence supporting the main effect of exercise (BF_10_ = 1.918), and no evidence supporting the main effect of time (BF_10_ = 0.521). Moderate evidence supporting the exercise [moderate, very-light] by time [baseline, post-exercise, post-cTBS] interaction (BF_10_ = 3.673) was apparent. To assess the evidence supporting the interaction term the Bayes Factor for the model with the interaction term was divided Bayes Factor for the model with only the main effects (exercise and time; 3.673/1.084). The interaction model was preferred over the main effects model by a Bayes Factor of 3.39, indicating there was moderate evidence supporting the inclusion of the interaction term over the main effects.

In the moderate exercise condition, the evidence supporting the main effect of time was extremely strong (BF_10_ = 0.6.85). *Post hoc* comparisons revealed extremely strong evidence supporting the contention that post-exercise interference scores were lower than baseline scores (BF_10_ = 6.44). Likewise, results indicated that there was strong evidence (BF_10_ = 9.31) supporting the notion that post-cTBS scores differed from baseline. There was no evidence suggesting that post-exercise and post-cTBS interference scores were different (BF_10_ = 0.263). Conversely, there was no evidence supporting a time effect in the movement-only control condition (BF_10_ = 0.261).

### ERP Results

#### P3b Amplitude

**FIGURE 3:**
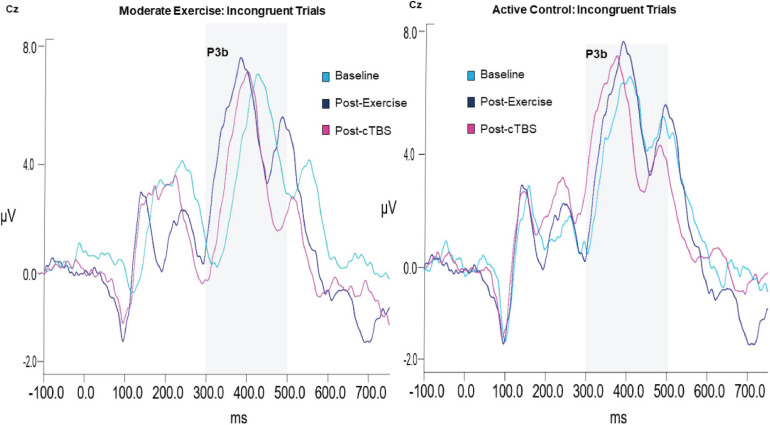
Grand average waveform of P3b ERP component.

Analysis of the P3b amplitude revealed a significant main effect of congruency (*F*(1,21) = 40.003, *p* < 0.001, *d* = 2.762). Across exercise conditions, the amplitude to incongruent trials was significantly larger than congruent trials; see Table 3 The main effects of exercise condition (*F*(1,21) = 1.85, *p* = 0.189, *d* = 0.594) and time (*F*(2,42) = 1.04, *p* = 0.363, *d* = 0.444), and exercise by congruency interaction (*F*(1,21) = 1.32, *p* = 0.263, *d* = 0.501) were not significant. Trends toward significance were observed for the exercise by time (*F*(2,42) = 2.86, *p* = 0.069, *d* = 0.739) and time by congruency (*F*(2, 42) = 2.80, *p* = 0.072, *d* = 0.732) interactions. The three-way interaction was not significant (*F*(2, 42) = 0.132, *p* = 0.876, *d* = 0.155; see Figure 3).

**TABLE 3:** P3b ERP Amplitudes (μV) and Latencies (ms) Across Condition and Time.

P3b	Moderate Intensity			Movement-only control		
	Baseline (SD)	Post-exercise (SD)	Post-cTBS (SD)	Baseline (SD)	Post-exercise (SD)	Post- cTBS (SD)
**Amplitude**						
Incongruent	7.12 (1.64)	7.42 (2.30)	7.13 (1.84)	5.89 (3.11)	7.45 (2.57)	6.44 (3.40)
Congruent	5.56 (1.62)	5.02 (1.65)	5.51 (1.27)	4.41 (1.72)	5.45 (1.75)	5.31 (2.23)
**Latency**						
Incongruent	423.75 (42.86)	373.78 (21.85)	390.41 (42.01)	432.10 (47.91)	412.45 (49.04)	378.62 (41.31)
Congruent	413.30 (51.15)	401.18 (45.14)	395.85 (34.30)	401.12 (50.05)	413.89 (54.77)	398.94 (59.13)

#### P3b Latency

Examination into the effects on P3b latency revealed a significant main effect of time (*F*(2, 42) = 8.36, *p* = 0.001, *d* = 1.263). The main effects of exercise condition (*F*(1, 21) = 0.84, *p* = 0.370, *d* = 0.398) and congruency (*F*(1, 21) = 0.84, *p* = 0.369, *d* = 0.398) were not significant. This was qualified by significant time by exercise (*F*(2, 42) = 5.28, *p* = 0.009, *d* = 1.003) and time by congruency (*F*(2,42) = 4.96, *p* = 0.012, n2 = 0.191) interactions. However, the three-way [time x exercise condition x congruency] interaction (*F*(2, 42) = 037, *p* = 0.963, *d* = 0.090) was not significant.

In the moderate exercise condition, results from the 2 × 3 [congruency by time] ANOVA revealed a significant time by congruency interaction (*F*(2,42) = 3.37, *p* = 0.044, *d* = 0.800). Similar to the behavioral results, a significant time effect was apparent for incongruent trials (*F*(2, 42) = 16.720 *p* < 0.001, *d* = 1.784). Specifically, compared to baseline (*M* = 423.75, *SE* = 9.14). P3b latencies were significantly shorter (*p* < 0.001, 95% CI [33.58, 66.36]) following a bout of moderate intensity exercise (*M* = 373.78; *SE* = 4.67). Additionally, post-cTBS (*M* = 390.41; *SE* = 8.96) latencies were marginally higher than those observed post-exercise (post-exercise; *p* = 0.052, 95%CI [−0.18, 33.44). Importantly, post-cTBS latencies were significantly shorter (*p* = 0.004, 95% CI [12.04, 54.64]) than baseline. No significant time effects were observed for congruent trials (*F*(2,42) = 1.004,*p* = 0.375, *d* = 0.440).

In the movement-only control condition, the main effects of time (*F*(2,42) = 6.13, *p* = 0.005, *d* = 1.08) and congruency (*F*(2,42) = 8.48, *p* = 0.008, d = 1.27) were significant. However, the congruency by time interaction was not significant (*F*(2,42) = 1.86 *p* = 0.168, *d* = 0.594). Across timepoints, congruent trials had significantly shorter latencies than incongruent trials (*p* = 0.008 95% CI [4.75, 28.50]). In addition, across congruency types, post-cTBS latencies were significantly shorter than those observed post-exercise (*p* = 0.003, 95% CI [9.46, 39.32]) and at baselin*e* (*p* = 0.006, 95% CI [8.79, 46. 87]). There were no significant differences observed when comparing post-exercise and baseline latencies (*p* = 0.720, 95% CI [−16.30, 23.19]).

#### N2 Amplitude

**FIGURE 4:**
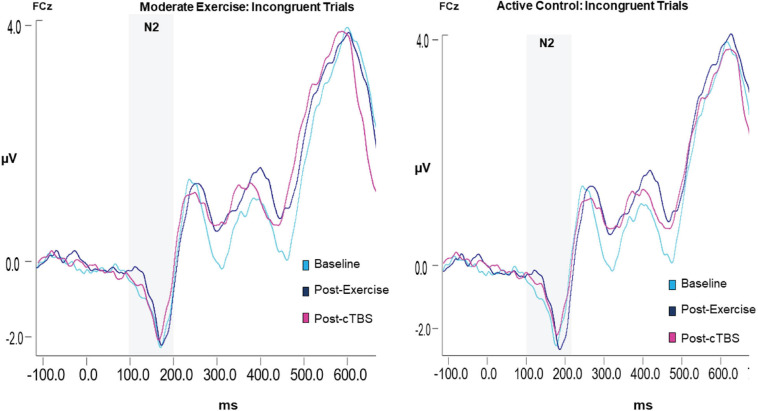
Grand average waveform of the N2 ERP component.

Results revealed a significant main effect of congruency (*F*(1,21) = 13.88, *p* = 0.001, *n*2 = 1.626), such that across exercise conditions and time points the N2 amplitude to congruent trials (*M* = −1.65, *SE* = 0.09) was smaller than that to incongruent trials (*M* = −2.25; *SE* = 0.19); see Table 4. The main effects of exercise condition (*F*(1, 21) = 0.250, *p* = 0.622, *n*2 = 0.012) and time (*F*(2,42) = 0.423, *p* = 0.658, *d* = 0.286). No significant interactions were observed (*p* > 0.20; see Figure 4).

**TABLE 4:** N2 ERP amplitudes (μV) and latencies (ms) across condition and time.

N2	Moderate Intensity			Movement-only control		
	Baseline	Post-exercise	Post-cTBS	Baseline	Post-exercise	Post-cTBS
**Amplitude**						
Incongruent	−2.11 (1.87)	−2.17 (1.08)	−2.02 (1.59)	−2.39 (1.75)	−2.67 (1.84)	−2.16 (1.61)
Congruent	−1.32 (1.60)	−1.59 (1.12)	−1.20 (1.01)	−1.77 (1.04)	−2.32 (0.72)	−1.70 (0.99)
**Latency**						
Incongruent	179.49 (69.35)	175.40 (59.62)	164.30 (73.24)	170.32 (62.73)	185.67 (71.29)	170.38 (69.46)
Congruent	158.75 (61.94)	158.40 (53.45)	160.81 (68.14)	162.10 (67.40)	168.56 (61.97)	168.48 (67.75)

#### N2 Latency

No significant main effects (*p* > 0.10) or interactions (*p* > 0.20) were observed when examining N2 latencies.

### Discussion

This investigation sought to assess the capacity of aerobic exercise to blunt the suppressive effects of cTBS targeting the left dlPFC. As expected, findings revealed an exercise effect in the moderate exercise condition, as evidenced in the Flanker task and P3b ERP components. Additionally, the results from the movement only control condition indicated no post-movement effects on cognitive control indices. Interestingly, evidence of a buffering effect of both aerobic exercise and light intensity movement on cTBS-induced attenuation was observed; however, given that the current study lacked a no-movement control condition, it is difficult to ascertain the effectiveness of cTBS in this study sample.

Collectively, the findings from this study suggest that moderate intensity exercise may provide a protective buffer against temporary perturbations in dlPFC functionality, and by extension top-down attentional control. Behaviorally, significant improvements in Flanker task performance were observed following an acute bout of moderate intensity exercise. Most notably, post-cTBS interference scores did not differ from post-exercise scores, but instead remained significantly lower compared to baseline, indicating a buffering effect of moderate intensity exercise on executive functions. Counter to our expectations, there were no significant differences in post-exercise and post-cTBS interference scores in the movement-only control condition. While this effect was unexpected, it may suggest that the intensities of both moderate aerobic exercise and the movement-only control condition may be sufficient to offset the temporary cTBS induced attenuation to cognitive control, given that cTBS reliably suppresses function within the dlPFC under usual circumstances (Lowe et al., 2018b). Although cTBS has been shown to reliably attenuate executive function, these findings should not take the place of a true control condition. Examination of neuroelectric measures revealed shorter P3b latencies to incongruent trials following moderate intensity exercise, suggesting faster cognitive processing speed post-exercise. Similar to the behavioral data, there were no significant differences in post-exercise and post-cTBS latencies. While the movement-only control condition revealed no neuroelectric changes post exercise, P3b latencies were significantly shorter following cTBS.

Taken together, the current findings add to the large literature describing effects of aerobic exercise on cognitive control, yet the lack of a true control condition warrants further investigation into the buffering effects of exercise on EF. Novel to the present investigation, the moderate intensity exercise session seemed to provide a protective buffer against experimentally induced perturbations in cognitive control; that is the ability of exercise to reduce the initial suppressive effect of cTBS. This expands on the findings by Lowe et al. (2017) wherein 28 female participants received cTBS prior to engaging in acute exercise. Consistent with Lowe et al., 2017, the current study demonstrated an exercise effect in the moderate intensity condition, such that behavioral scores significantly improved following exercise. Lowe et al., 2017 employed a Stroop task as a behavioral measure of cognitive control, however given that ceiling effects of accuracy that were observed (> 0.97), the current study employed a Flanker paradigm, resulting in lower accuracy scores (< 0.86).

Further, Lowe et al. (2017), observed experimentally induced attenuation following cTBS in both conditions, along with a strong recovery effect following moderate intensity exercise, suggesting a protective and recovery mechanism against suppression to the dlPFC. Within the current study, it is unclear whether cTBS was successful in supressing cognitive function, as a post-stimulation effect was not evidenced. Consistent with our hypothesis, behavioral task scores in the moderate intensity exercise condition did not significantly worsen following cTBS stimulation, however surprisingly, the same phenomenon was observed in the movement only condition, suggesting a possible buffering effect of both aerobic exercise and light intensity movement.

These findings have substantial implications for how we conceptualize the beneficial effects of aerobic exercise on brain health and executive control. That is, while the current study employed an experimental perturbation (specifically, cTBS), there are many naturally occurring perturbations in everyday life, including lack of sleep, mood fluctuations, and acute stress (Fossati et al., 2002; Nilsson et al., 2006; Porcelli and Delgado, 2009; Tucker et al., 2010; Shields et al., 2016). If generalizable to these types of everyday perturbations, the current findings suggest the possibility that exercise may serve to provide protection against momentary fluctuations in cognitive control in everyday living. Such findings may have implications for clinical intervention strategies as well. For instance, acute bouts of exercise may provide an optimal intervention or preventative strategy for people who are chronically subject to exposure to lifestyle perturbations mentioned previously (e.g., shift workers, hospital employees). Additionally, given that buffering effects also seem to manifest in relation to light intensity movement, the effects of this intervention could extend to individuals who may not be physically able to perform moderate intensity exercise, such as older adults.

While significant behavioral effects were apparent in the moderate intensity exercise condition, this did not manifest as changes in P3b amplitudes as expected, or N2 amplitudes. However, significant P3b latency effects to incongruent trials were observed. Consistent with the behavioral data, shorter latencies were apparent following the bout of moderate intensity exercise. Further, post-cTBS P3b latencies were significantly shorter than baseline in the moderate exercise condition. In general, the amplitude of the P3b and N2 ERP components are thought to reflect the neural resources afforded to a given task, whereas, the latency is thought to be an index of cognitive processing speed or the speed at which an individual is able to classify and evaluate a stimulus (Polich, 2007; Folstein and Van Petten, 2008). Therefore, results from the current study seem to suggest that the buffering effects of exercise are specific to cognitive processing speeds rather than attentional allocation or conflict adaptation *per say*, a finding that is consistent with several other studies. For instance, P3b latencies in both young and older adults have been shown to be shorter following both light and moderate intensity exercise (Kamijo et al., 2007, 2009).

Additional evidence supporting this notion comes from rTMS prior studies utilizing neuroelectric measures of cognitive control. Stimulation-induced reductions in the latency of the P3b component is observed following both 10Hz and 20Hz (i.e., excitatory stimulation) rTMS targeting the left PFC (Evers et al., 2001; Rektor et al., 2010; Pinto et al., 2018). Likewise, several studies using inhibitory rTMS or cTBS targeting the dlPFC have reported that attenuation of left dlPFC activity results in the subsequent increase in P3b latencies (Evers et al., 2001; Jing et al., 2001; Hansenne et al., 2004; Pinto et al., 2018). Further, recent findings demonstrated that stimulation-induced increases in P3b latencies to incongruent flanker trials are observed following cTBS over the left dlPFC (Lowe et al., 2018b). These findings are noteworthy, as the same stimulation, behavioral task, and EEG protocols were employed as in the current study. Assuming that cTBS was effective in this sample, these data demonstrate that P3b latencies are sensitive to rTMS-induced attenuation of dlPFC activity, indicating that moderate intensity exercise as well as light intensity movement does indeed provide a protective buffer against cTBS-induced perturbations to executive control. Further, evidence exists to suggest that high-fit individuals outperform low-fit counterparts on cognitive tasks and demonstrate faster processing speeds (Hillman et al., 2005; Dupuy et al., 2015). This may provide an alternate explanation for these findings; such that the current study recruited a high-fit group who exercised frequently, and as a result preserved an exercise buffer, resulting in better than expected Flanker interference scores and P3b latencies. It is possible however that cTBS was not effective in the current study sample, as it remains unclear as to whether these findings were indeed buffering effects or merely a carry-over effect of exercise, due to the lack of a non-movement control.

Exercise-induced improvements in inhibitory control are thought to be mediated, at least in part, by increased cerebral blood flow to the left dlPFC (Yanagisawa et al., 2010; Endo et al., 2013; Byun et al., 2014; Giles et al., 2014; Guiney et al., 2015). Considering the association between aerobic exercise and increased cerebral blood oxygenation, it is possible that both light and moderate intensity exercise are sufficient to offset cTBS- induced attenuation to neuronal populations within the left dlPFC. It is also possible that elevated levels of certain neurotransmitters such as norepinephrine, epinephrine, serotonin, and dopamine, induced a buffering effect, as they have been demonstrated to be elevated following acute bouts of exercise. ERP latencies and amplitudes are thought to be influenced by dopaminergic function, subsequently impacting cognitive processing speeds and neural resources allocated to a specific task (Ullsperger et al., 2014). There is increasing evidence to suggest that light intensity exercise may also result in increased cerebral blood oxygenation (Byun et al., 2014). For instance, previous studies have indicated that light intensity walking increases cerebral blood oxygenation to the PFC (Suzuki et al., 2004; Holtzer et al., 2011; Matsukawa et al., 2020). Additionally, movement only conditions such as yoga have been shown to sufficiently improve cognitive task scores as well as mood scores (Berger and Owen, 1988; Gothe et al., 2013). Furthermore, studies have suggested that specific self-efficacy combined with positive mood may improve scores on executive control tasks (Berger and Owen, 1992). According to the POMS 2-A mood scores in Appendix Figure 3 of the Appendix, it appears that mood disturbance scores improved from baseline following the movement only condition. However, the buffering effects of exercise have been largely unexplored and may differ from other proposed mechanisms. Further research is warranted to better determine the neurophysiological processes underlying cortical buffering.

Strengths in the present study include the use of a within-subject study design in an effort to minimize any inter-individual variability. Additionally, the use of cTBS as our neuromodulation protocol provided a safe and reliable method by which to investigate the buffering effects of exercise. Limitations of the present study include sampling a healthy university student population, who may not be as receptive to the effects of exercise compared to an older adult sample due to high initial levels of cognitive performance. Previous literature has suggested that older adults tend to demonstrate greater performance improvements compared to young healthy adults following acute bouts of exercise (Chang et al., 2012a; during the rest period participants were asked to sit comfortably for 10 min. Although both the moderate intensity exercise and movement-only control groups appeared to demonstrate a potential buffering effect, this cannot be known definitively in the absence of a no-movement control condition. Without this, an alternative interpretation of the findings is that no cTBS effect emerged in either condition, which is possible given that not all studies show significant perturbation effects on executive function following cTBS. Future studies should aim to disentangle this issue.

### Conclusion

Although some of the results of the current study are unexpected, these findings can provide some initial evidence that moderate intensity exercise may blunt the usual suppressive impact of cTBS on the dlPFC. These findings may further suggest that very low intensity movement also provide some protection against cTBS induced suppression. However, due to the lack of a no-movement control group, it is difficult to ascertain whether cTBS was indeed effective in this target population. Additional research on the buffering effect of exercise using natural suppressors—such as sleep deprivation, alcohol consumption, acute stress—may be potentially useful. Likewise, exploring the reliability of buffering effects of exercise in other populations may be beneficial, and could provide a more comprehensive understanding of how exercise benefits the brain in everyday life.

## Data Availability Statement

The raw data supporting the conclusions of this article will be made available by the authors, without undue reservation.

## Ethics Statement

The studies involving human participants were reviewed and approved by University of Waterloo Research Ethics Board. The patients/participants provided their written informed consent to participate in this study.

## Author Contributions

FM and CL conceived the study, collected and analyzed the data, and wrote the manuscript. Both authors contributed to the article and approved the submitted version.

## Conflict of Interest

The authors declare that the research was conducted in the absence of any commercial or financial relationships that could be construed as a potential conflict of interest.

## Publisher’s Note

All claims expressed in this article are solely those of the authors and do not necessarily represent those of their affiliated organizations, or those of the publisher, the editors and the reviewers. Any product that may be evaluated in this article, or claim that may be made by its manufacturer, is not guaranteed or endorsed by the publisher.
